# Green under siege: how the polyhexamethylene guanidine disinfectant attacks microalgae in aquatic ecosystems

**DOI:** 10.7717/peerj.19553

**Published:** 2025-06-11

**Authors:** Kai Zhang, Ziqin Wei, Yixuan Wang, Weikai Shui, Bingchan Jia, Zhuo Huang, Qian Feng

**Affiliations:** 1 Nanjing Jiangning District Water Affairs Bureau, Nanjing, China; 2 Nanjing No. 1 Middle School, Nanjing, China; 3 High School Affiliated to Nanjing Normal University Jiangning Campus, Nanjing, China; 4 College of Environment, Hohai University, Nanjing, China; 5 Hohai University, Key Laboratory of Integrated Regulation and Resource Development on Shallow Lakes, Ministry of Education, Nanjing, Jiangsu, China; 6 Center for Science and Technology Achievement Promotion, Yangtze River Scientific Research Institute, Wuhan, China; 7 Wuhan Changjiang Kechuang Technology Development Co., Ltd., Wuhan, China

**Keywords:** Polyhexamethylene guanidine (PHMG), Microalgae, Photosynthesis, Antioxidative stress, Structural equation model (SEM)

## Abstract

In aquatic ecosystems, microalgae experience the adverse effects of the widely used disinfectant component, polyhexamethylene guanidine (PHMG). Our research focused on the model alga, *Chlorella vulgaris*, examining how PHMG exposure influences its growth, photosynthesis, metabolic byproducts, and antioxidant defenses. We observed a range of effects from slight disturbances at low PHMG concentrations to significant growth inhibition at higher levels, suggesting a hormesis-like response. Specifically, PHMG exposure led to reduced chlorophyll content, impaired Photosystem II efficiency, and decreased photosynthetic activity. Interestingly, the algae responded to stress by increasing antioxidant enzyme activities and stress biomarkers. Structural equation modeling (SEM) revealed that PHMG primarily disrupts the photosynthetic apparatus, which in turn affects metabolic and antioxidant responses, culminating in reduced algal biomass. Our results contribute to the understanding of the threats posed to aquatic life by the ubiquitous and ever-increasing pollution from chemical disinfectants, and highlight the urgency of mitigation measures.

## Introduction

Polyhexamethylene guanidine (PHMG) is a third-generation guanidine bactericide that is extensively utilized in medical, sanitary, cleaning, and various other industries due to its broad-spectrum antibacterial efficacy, prolonged bacteriostatic activity, strong antimicrobial properties, and low toxicity and side effects (Zhang, Jiang & Chen, 1999; Walczak, Richert & Burkowska-But, 2014; Kim et al., 2018). In 2017, China produced 56,000 tons of guanidine bactericides. The COVID-19 epidemic has further accelerated the development and utilization of bactericides (Wang et al., 2022b). Survey data indicate that over the past 5 years, the annual growth rate of PHMG production has exceeded 16.67%. this increase in production and usage has hence led to considerable environmental concerns, primarily due to the excessive release and subsequent accumulation of PHMG in aquatic ecosystems, particularly affecting rivers and lakes (He et al., 2022; Wang et al., 2022b).

Microalgae, critical autotrophic components of these aquatic systems, are instrumental in maintaining ecological balance and resilience (Shi et al., 2021; Ma et al., 2022). They are frequently utilized to evaluate the ecological impact of novel contaminants, such as antibiotics, on river and lake systems, due to their high sensitivity to pollutants (Sendra et al., 2018; Yu et al., 2022). Recent studies have verified that PHMG bactericides could effectively inhibit bacterial growth at specific concentrations (Xiao et al., 2020; Peng et al., 2021). However, research on the ecological toxicity of PHMG to aquatic organisms, particularly microalgae, within river and lake ecosystems for ecological risk assessment remains insufficient (Poštulková & Kopp, 2016; Annenkov et al., 2020). Does the presence of PHMG in water impact the growth and metabolism of microalgae that are aquatic organisms? What are the potential mechanisms underlying these impacts? These issues necessitate additional research and further investigation. Polyhexamethylene guanidine (PHMG) is widely used as a biocide in chemical toilet additives for the management of faecal sludge. Disposal of this biocide-treated effluent to wastewater treatment plants is a major environmental concern. For example, large amounts of consumed PHMG can enter the wastewater and eventually accumulate in the waste activated sludge (WAS), which has potential implications for subsequent biological treatment of the effluent. While direct monitoring data of PHMG in natural water bodies remain limited, existing studies suggest its potential accumulation in aquatic systems through sludge disposal pathways. Guanidine derivatives (structural analogues of PHMG) have been detected in municipal wastewater treatment effluents, highlighting the persistence of such disinfectants in the aquatic environment (Wang et al., 2022b; He et al., 2022). These studies support the hypothesis that PHMG could enter and persist in aquatic systems, particularly in areas with intensive industrial or medical use of disinfectants.

The toxic effects of PHMG are not limited to lower organisms. Kim et al. (2018) found that PHMG induced pulmonary fibrosis in mammals, suggesting that it may be potentially risky for higher aquatic vertebrates such as fish. In addition, in a study exploring the potential toxic interactions of a binary mixture of PHMG with another disinfectant (benzylchloroammonium chloride, BEC) on Daphnia, a stronger antagonistic effect was found in the PHMG binary mixture, with a higher risk of contamination of the aquatic environment (Yang et al., 2023). Therefore, based on the safety of polyhexamethylene guanidine (PHMG), we hypothesize that different concentrations of PHMG may have varying impacts on the aquatic environment.

This study utilized PHMG, a commonly used bactericide that has gained popularity following the COVID-19 pandemic, as an external stressor. The focus was on *C. vulgaris*, a prevalent type of algae usually present in river and lake environments (Baltazar et al., 2014). The study examined how varying levels of PHMG exposure impacted the growth, metabolism, photosynthetic properties, and antioxidant system of *C. vulgaris*. Employing a structural equation model, we aimed to elucidate the response mechanisms of microalgae to PHMG exposure, thereby enhancing our understanding of the ecological toxicity of PHMG and its broader implications to aquatic life in rivers and lakes.

## Materials and Methods

### Algal species and culture experiment

The *C. vulgaris* strain (FACHB-25) utilized in this study was sourced from the Freshwater Algae Culture Collection at the Institute of Hydrobiology, Chinese Academy of Sciences. The experimental procedure involved cultivating and introducing *C. vulgaris* onto Blue-Green medium (BG11 medium), it was composed of 1.5 g/L NaNO_3_, 0.075 g/L MgSO_4_, 0.03 g/L K_2_HPO_4_, 0.036 g/L CaCl_2_, 0.006 g/L citric acid, 0.006 g/L ferric ammonium citrate, 0.001 g/L EDTA, 0.020 g/L Na_2_CO_3_, and 1 mL of a trace metal solution containing 2.86 g/L H_3_BO_3_, 1.81 g/L MnCl_2_, 0.22 g/L ZnSO_4_, 2.86 g/L NaMoO_4_, 0.08 g/L CuSO_4_, and 0.05 g/L Co(NO_3_)_2_ (Wang et al., 2023b). The BG11 medium was sterilized at 121 °C for 30 min in an autoclave, then cooled to room temperature in a laminar flow hood. The pH was subsequently adjusted to 7.1 using 1M NaOH and HCl solutions.

In a sterile setting, a predetermined quantity of *C. vulgaris* was introduced into the sterilized medium. The cultures were maintained at 25 °C under a light intensity of 2,000 Lux with a 12 h light-dark cycle. Upon reaching a specific cell density, the microalgae were then transferred to conical flasks at a ratio of 1:5 (*v*/*v*) with the medium. To prevent sedimentation of *C. vulgaris*, the conical flasks were shaken three times a day. Regular microscopic examinations were performed to monitor for contamination, and any detected contamination was addressed using the streak plate method. Once *C. vulgaris* reached the logarithmic growth phase (Luo & Zhao, 2024), it was prepared for experimental purposes.

### Experimental procedure

A blank control group (no PHMG added) was established, and PHMG was dissolved in milli Q water to prepare four different nominal concentrations of polyhexamethylene guanidine (PHMG) (0.03, 0.3, 0.6, and 3 mmol/L) as exposure concentrations for the algal bloom during the logarithmic growth period (Poštulková & Kopp, 2016). These concentrations were selected based on the concentrations of guanidine biocide products and emissions from major industrial activities (Wang, Yu & Dong, 2020; Wang et al., 2022a, 2022b, 2023a). The concentrations of PHMG chosen for the experiments (0.03–3 mmol/L) are much higher than the typical concentrations found in current natural waters, but are nevertheless relevant. The low concentration group (0.03 mmol/L) is close to the potential contamination level in the real environment, while the high concentration group (3 mmol/L) is used to assess the ecological risk under extreme conditions (Wang et al., 2022b; He et al., 2022). Meanwhile, PHMG (purity: ≥95%, (C_7_H_15_N_3_)n·xHCl) was bought from Sigma-Aldrich (St. Louis, MO, USA).

Various parameters of *C. vulgaris* were monitored throughout the experiment, including the biomass, extracellular polymeric substances (EPS), photosynthetic pigments, chlorophyll fluorescence parameters, and antioxidant enzymes, which aimed to elucidate the effects of PHMG on the growth, metabolism, photosynthetic characteristics, and antioxidant system of the algae.

Three replicates were established for each group to guarantee the reproducibility of the experimental data. The conical flasks were agitated three times a day, and their placement in the light culture box was frequently rotated to prevent uneven light dispersion.

The density of microalgae in the exponential growth phase was 1 × 10^6^ cells/mL. The algal culture medium was shaken three times a day and the algal cells were counted daily at 0, 24, 48, 72 and 96 h by hemocytometer with inverted microscope (Zhao et al., 2019).

An axenic culture of *C. vulgaris* was established by streaking algal samples on solid medium and incubating at 25 ± 1 °C under a 12 h:12 h light-dark cycle (2,000 Lx). After 7–10 days, colonies were isolated and confirmed as pure through microscopic examination (Yang et al., 2010; Wang et al., 2022c).

### Characterization analysis

#### Microalgal biomass

Optical density (OD_680 nm_) was used as a proxy for microalgae biomass using a UV spectrophotometer (UV1800PC; Xipu, Shanghai, China) (Ou et al., 2023). For each sample, a 50 mL amount was centrifuged at 4,000 rpm for 10 min using a high-speed chilled centrifuge, and the supernatant was discarded. The resulting pellet was then desiccated in a 60 °C oven for 24 h until a consistent weight was achieved, then cooled to room temperature and measured. The biomass *G* of microalgae was calculated using the following formula:

(1) $$G({\rm g/L) = }\displaystyle{{{W_1} - {W_2}} \over {V \times {{10}^{ - 3}}}}.$$

where *W*_1_ represents the weight of the centrifuge tube with algae (g), *W*_2_ represents the weight of the centrifuge tube (g), and *V* represents the sample volume (mL).

A calibration curve was generated correlating the OD680 of *C. vulgaris* with its biomass concentration in grams per liter. The linear regression analysis, conducted with Origin software, provided the correlation equation between the absorbance and biomass concentration.

(2) $$G\left( {g/L} \right) = 0.2461 \times O{D_{680}} - 0.0032,\; {R^2} = 0.9866.$$

#### The chlorophyll content of algal cells

The chlorophyll content of algal cells was quantified using the spectrophotometric technique (Russel et al., 2020). An algal solution (20 mL) with an optical density of OD_680 nm_ = 0.3 was centrifuged in a 50 mL centrifuge tube at 8,000 rpm for 10 min at 4 °C. The supernatant was discarded, and 2 mL of dimethyl sulfoxide was added to the tube. The mixture was then incubated in a water bath at 65 °C for 2 h. Then, 3 mL of 80% acetone was added to the centrifuge tube, thoroughly agitated, and the resulting liquid was separated and gathered in a light-protected centrifuge tube. Absorbance measurements were taken at wavelengths of 663 and 646 nm, with acetone serving as the blank.

The formulas used to compute the chlorophyll *a* and chlorophyll *b* concentrations were as below (Zhou et al., 2017):

(3) $$Ca{\rm (}{\rm mg}/{\rm L}{\rm )} = 12.21{A_{663}} - 2.81{A_{646}},$$

(4) $$Cb{\rm (}{\rm mg}/{\rm L}{\rm )} = 20.1{A_{663}} - 5.03{A_{646}}.$$

The variables were defined as follows: *Ca* = chlorophyll *a* content, *Cb* = chlorophyll *b* content, *A*663 = absorbance at 663 nm, and *A*646 = absorbance at 646 nm.

#### The maximum photochemical efficiency of Photosystem II (PS II)

The maximum photochemical efficiency of Photosystem II (PS II) was determined by measuring *F*v/*F*m using the chlorophyll fluorescence technique (Schreiber, Schliwa & Bilger, 1986; Genty, Briantais & Baker, 1989; Harbinson, Genty & Baker, 1989; Ryan, Ralph & McMinn, 2004).

The fluorescence analysis of microalgae was performed using a portable chlorophyll fluorescence meter (AquaPen-C AP-C 100, Photo Subsystem Instrument, Czech Republic) according to previously reported methods (Markou & Muylaert, 2016). An algal solution (2 mL) was added to a colorimetric dish and placed into a phytoplankton fluorometer. Following a 15 min period of dark adaption, measurements were conducted using the AquaPen program.

(5) $$\displaystyle{{{F_v}} \over {{F_m}}} = \displaystyle{{{F_m} - {F_0}} \over {{F_m}}}.$$

where *F*_0_ is the minimum fluorescence yield in the dark-adapted state, while *F*_m_ is the maximum fluorescence yield measured after the first saturating flash under the dark-adapted condition (Genty, Briantais & Baker, 1989).

#### The contents of EPS

EPS produced by *C. vulgaris* were extracted using the method described by Ou et al. (2023). Polysaccharides in EPS were measured using the anthrone-sulfuric acid technique (Raunkjær, Hvitved-Jacobsen & Nielsen, 1994), whereas proteins were quantified using the Lowry method (Frølund et al., 1996).

#### Antioxidant enzyme activities

Antioxidant enzyme activities were evaluated by measuring the superoxide dismutase (SOD) activity with the nitroblue tetrazolium (NBT) photochemical reduction method (Chen et al., 2016), catalase (CAT) activity using UV spectrophotometry (Chen et al., 2016), and malondialdehyde (MDA) levels with the enzyme-linked immunosorbent assay (ELISA) method (Yu et al., 2024).

#### Statistical analysis

An analysis of variance (ANOVA) with a least significant difference test was used to assess the significance of the results, and *p* < 0.05 was considered statistically significant.

Structural equation modeling (SEM) analysis using Smart PLS on the contribution of diverse factors to the destiny of Biomass and PHMG (SEM is a statistical analysis technique that includes factor analysis and path analysis, which is applicable to the study of interrelationships among multiple variables) (Han et al., 2020). Meanwhile, all experiments were conducted in triplicate. Path coefficients and coefficients of determination (R^2^) were calculated after 999 bootstraps with significance levels reported as *p* < 0.05* and *p* < 0.001*** (Wang et al., 2024).

SEM is a statistical technique employed to investigate the relationships between variables through their covariance matrix, playing a pivotal role in the analysis of multivariate data (Amini & Alimohammadlou, 2021). The author used SEM to analyze the relationships and pathways among the PHMG exposure, growth, metabolism, photosynthetic characteristics, and antioxidant system in microalgae.

## Results

### Microalgae growth curve

**Figure 1:**
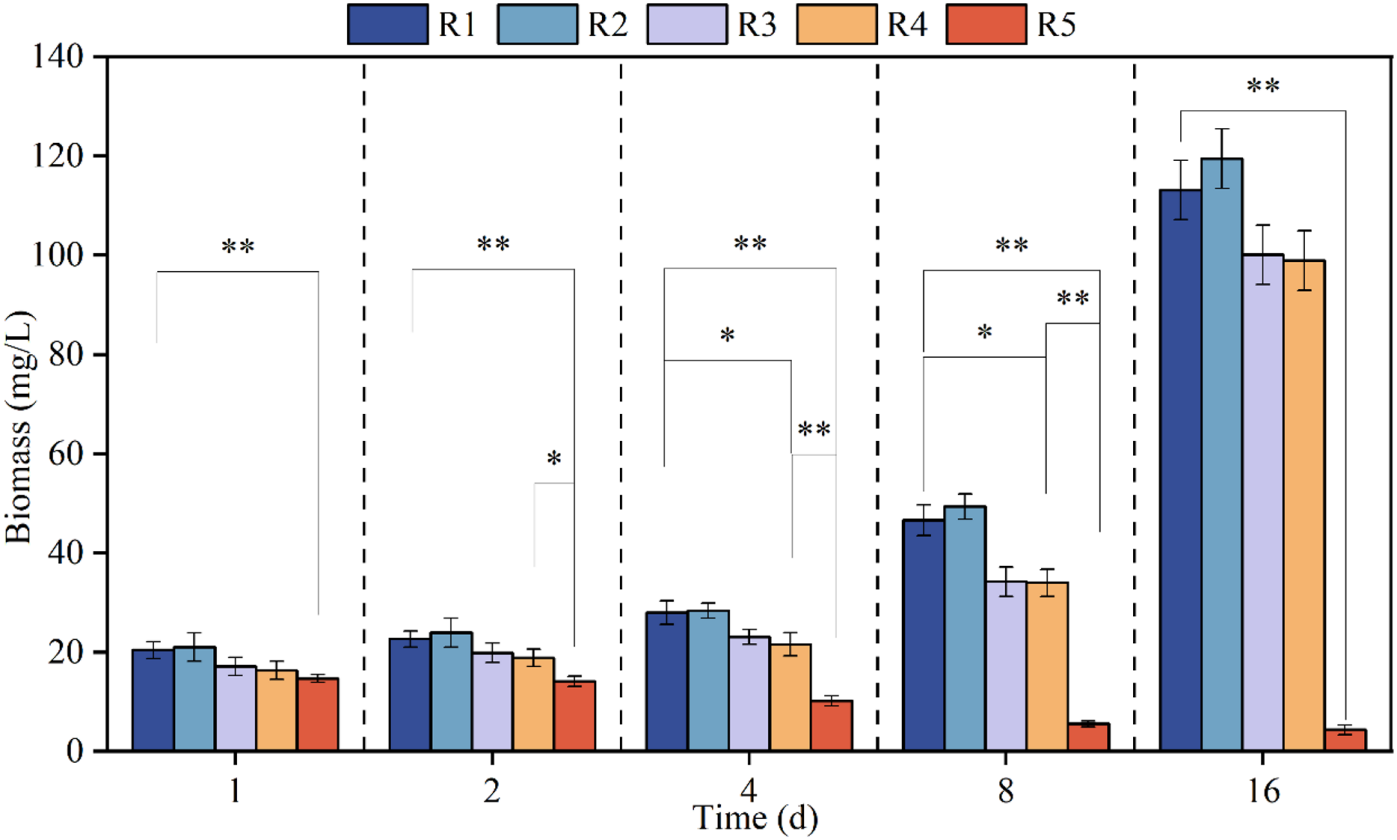
The growth curve of *Chlorella vulgaris* when exposed to various concentrations of PHMG. *p < 0.05, **p < 0.01. R1: A blank control group (no PHMG added), R2: PHMG exposure concentration 0.03 mmol/L, R3: PHMG exposure concentration 0.3 mmol/L, R4: PHMG exposure concentration 0.6 mmol/L, R5: PHMG exposure concentration 3 mmol/L.

Figure 1 shows the growth curve of microalgae when exposed to different concentrations of PHMG for different time intervals, namely 1, 2, 4, 8 and 16 days. Microalgae biomass decreased steadily through time when exposed to 3 mmol/L PHMG; however, for the other reactors, the algal biomass progressively increased. Compared to the stable growth in the control group, the biomass marginally increased overall under 0.03 mmol/L PHMG exposure, but the increment was less than 5%. When the PHMG exposure was increased to 0.3 and 0.6 mmol/L (R3 and R4), the biomass continued to increase, but there was noticeable growth inhibition compared to the blank control group. By Day 16, the biomass in R3 and R4 decreased from 113.1 mg/L in the blank control group (R1) to 100.1 and 98.9 mg/L, respectively, representing the reductions of 11.5% and 12.6%.

The biomass results of the higher concentration group (>0.3 mmol/L) and the control group were analyzed using ANOVA analysis and it was found that the higher concentration of PHMG exposure (R5) reduced the biomass of the microalgae very significantly (*p* < 0.01) throughout the experimental cycle. The higher concentration of PHMG exposure (R4) also caused a significant reduction in microalgal biomass on days 4 and 8 of the experiment (*p* < 0.05).

### Mechanism of photosynthesis in microalgae

**Figure 2:**
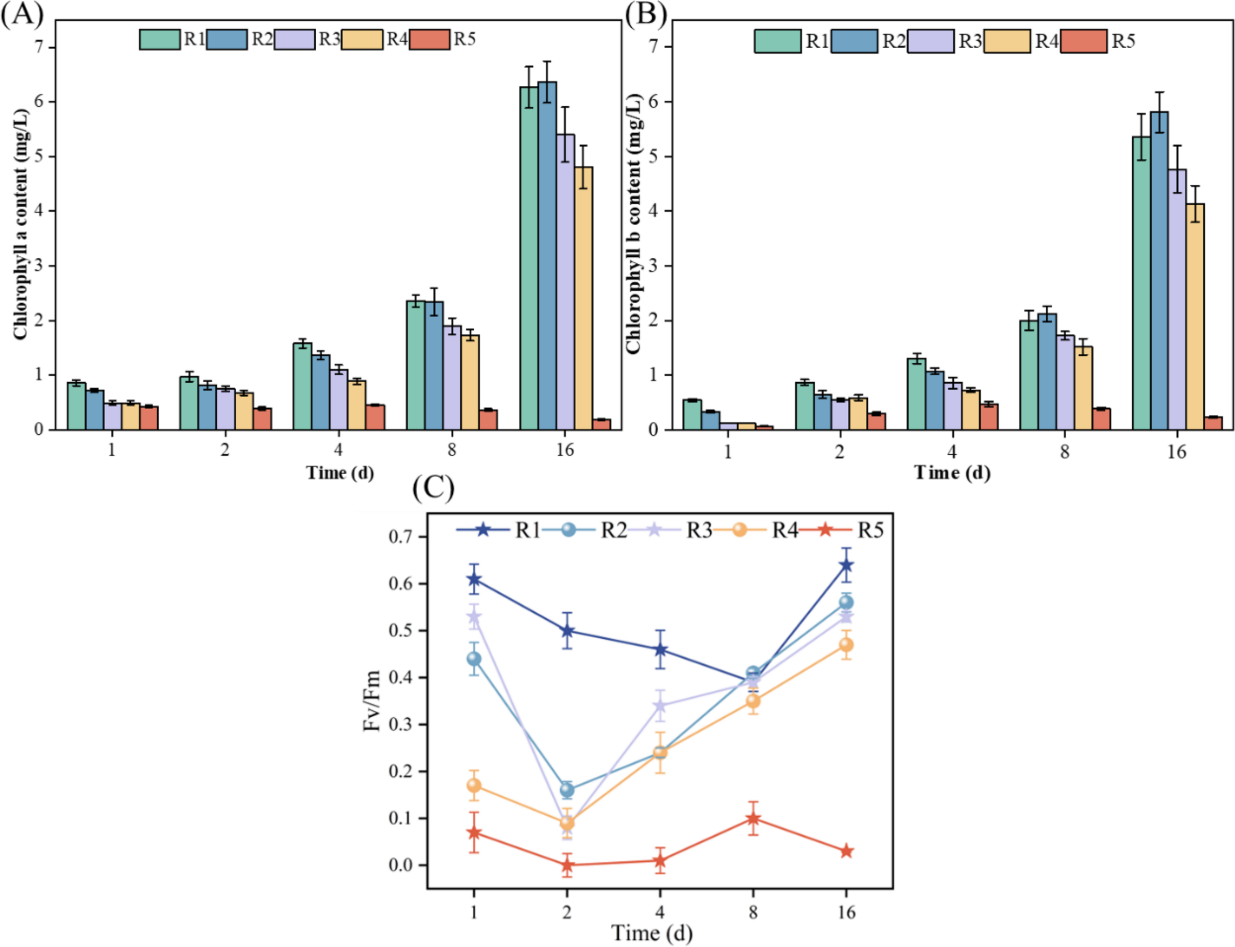
Impacts of various PHMG concentrations on the photosynthetic mechanism of microalgae. (A) Chlorophyll a content. (B) Chlorophyll b content. (C) Maximal photochemical efficiency of PS II. R1: A blank control group (no PHMG added), R2: PHMG exposure concentration 0.03 mmol/L, R3: PHMG exposure concentration 0.3 mmol/L, R4: PHMG exposure concentration 0.6 mmol/L, R5: PHMG exposure concentration 3 mmol/L.

As illustrated in Figs. 2A and 2B, during the cultivation period, microalgae experienced a decrease in chlorophyll *a* from 0.42 mg/L on Day 1 to 0.19 mg/L on Day 16 when exposed to a high concentration (3 mmol/L) of PHMG (R5). Conversely, under the other conditions tested, both chlorophyll *a* and chlorophyll *b* levels in microalgae consistently increased. When exposed to 0.03 mmol/L of PHMG, microalgal cells initially produced lower levels of chlorophyll *a* and chlorophyll *b* compared to the control group from Days 1 to 4. However, from Days 8 to 16, the levels of both chlorophyll *a* and chlorophyll *b* recovered to levels comparable to those of the control group. On Day 4, chlorophyll *a* and chlorophyll *b* levels were 1.4 and 1.1 mg/L, respectively, following the exposure to 0.03 mmol/L PHMG, representing a 12.5% and 15.4% decrease compared to the control group’s chlorophyll *a* content of 1.6 mg/L and chlorophyll *b* content of 1.3 mg/L. By Day 16, the chlorophyll a and chlorophyll b contents increased from 6.2 and 5.3 mg/L in the control group to 6.4 and 5.8 mg/L, respectively.

Figure 2C demonstrates the simultaneous effect of different PHMG concentrations on the maximal photochemical efficiency of PS II. Compared to the control group, exposure to PHMG concentrations ranging from 0.03 to 0.60 mmol/L resulted in a decrease in Fv/Fm to the lowest value on day 2, followed by a gradual increase. In contrast, exposure to 3 mmol/L of PHMG maintained Fv/Fm at a consistently low level. Additionally, the maximum photochemical efficiency of PS II at PHMG concentrations ranging from 0.03 to 0.60 mmol/L was significantly lower than that of the control group without PHMG. Higher concentrations of PHMG were associated with lower Fv/Fm ratios.

### Microalgal EPS composition

**Figure 3:**
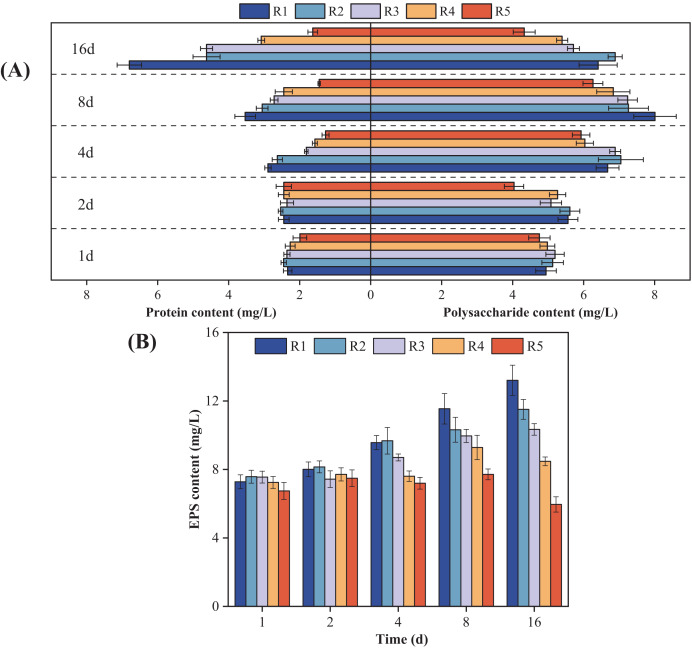
Changes in the microalgal EPS composition under various PHMG concentrations. (A) Protein and polysaccharide content. (B) EPS content. R1: A blank control group (no PHMG added), R2: PHMG exposure concentration 0.03 mmol/L, R3: PHMG exposure concentration 0.3 mmol/L, R4: PHMG exposure concentration 0.6 mmol/L, R5: PHMG exposure concentration 3 mmol/L.

Figure 3 shows how the microalgal EPS components changed over time when exposed to different concentrations of PHMG. Compared to the control group, the protein content in microalgal cells exposed to 0.03 mmol/L PHMG consistently increased over time. However, for PHMG concentrations of 0.3 to 0.6 mmol/L, the protein content decreased to the minimum value by Day 4 and then gradually increased. Exposure to 3 mmol/L of PHMG resulted in the protein content remaining at a consistently low level. Additionally, for microalgal cells exposed to PHMG concentrations ranging from 0.03 to 0.6 mmol/L, the polysaccharide content followed a similar trend to that of the control group, initially increasing and then decreasing. In contrast, exposure to 3 mmol/L of PHMG caused the polysaccharide content to reach the minimum value by Day 2, followed by a peak on Day 8 before decreasing again. Overall, during the early exposure period (Days 1–2), EPS secretion showed minimal changes compared to the control group. However, by Day 4, a significant decrease in EPS secretion was observed. On Day 16, the EPS components in microalgal cells changed: the control group had 6.4 mg/L polysaccharides and 6.8 mg/L proteins, while the experimental groups showed different levels: R2 had 6.9 mg/L polysaccharides and 4.6 mg/L proteins, R3 had 5.7 mg/L polysaccharides and 4.6 mg/L proteins, and R4 had 5.4 mg/L polysaccharides and 3.1 mg/L proteins.

### The antioxidant system

**Figure 4:**
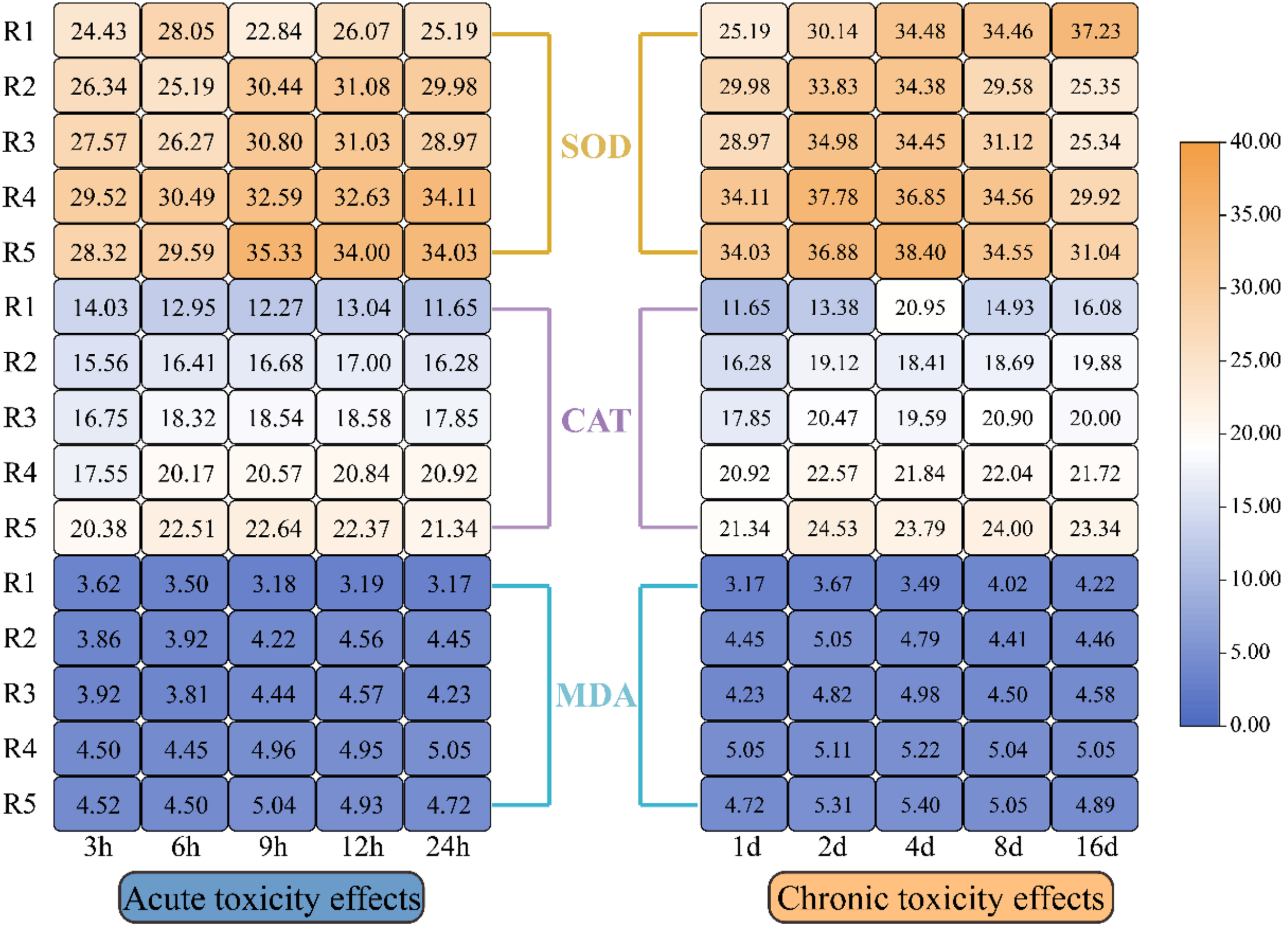
Impacts of various PHMG concentrations on the antioxidant system of microalgae. (R1: A blank control group (no PHMG added), R2: PHMG exposure concentration 0.03 mmol/L, R3: PHMG exposure concentration 0.3 mmol/L, R4: PHMG exposure concentration 0.6 mmol/L, R5: PHMG exposure concentration 3 mmol/L).

Figure 4 illustrates how oxidative stress indicators in microalgae responded to different levels of PHMG exposure. The data revealed a progressive rise in CAT, SOD, and MDA levels in microalgal cells with the rising PHMG concentration and extended exposure time. While EPS showed a small reaction during the first 1–2 days, significant changes in oxidative stress indicators were observed within 3–6 h of treatment compared to the control group. The magnitude of these variations diminished as the exposure duration extended from 4 to 16 days.

### Impacts of PHMG exposure on different parameters of microalgae

**Figure 5:**
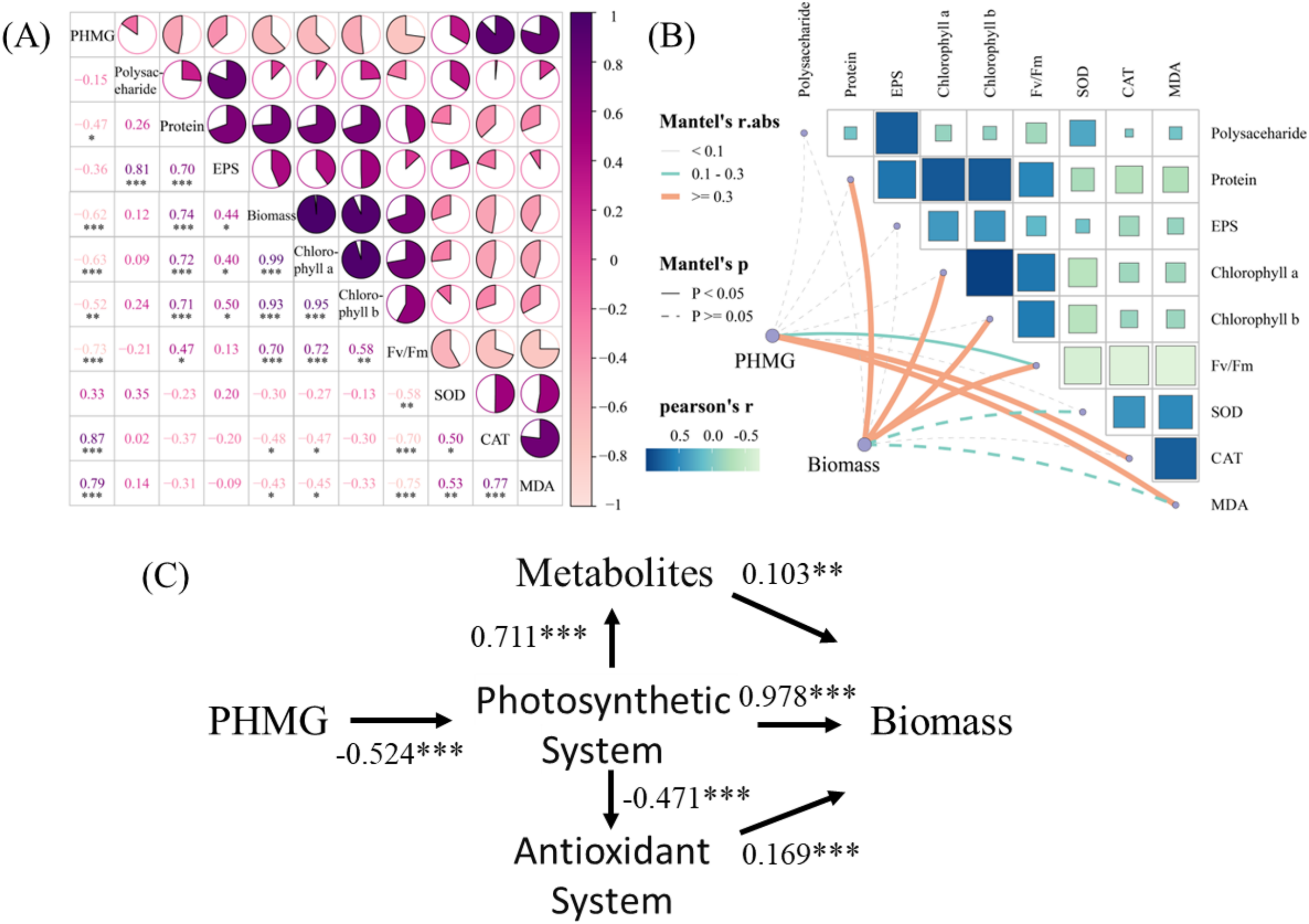
Impacts of PHMG exposure on different parameters of microalgae. (A) The correlation analysis of parameters in microalgae. (B) The Mantel’s test examines the correlation among the PHMG exposure concentration, biomass, and several parameters of algal cells. R1: A blank control group (no PHMG added), R2: PHMG exposure concentration 0.03 mmol/L, R3: PHMG exposure concentration 0.3 mmol/L, R4: PHMG exposure concentration 0.6 mmol/L, R5: PHMG exposure concentration 3 mmol/L. Asterisks indicate statistical significance at *p < 0.05, **p < 0.01, and ***p < 0.001.

The correlation analysis (Fig. 5A) revealed significant positive correlations between PHMG exposure and the secretion of SOD (r = 0.87) and MDA (r = 0.79) in microalgae. Conversely, PHMG exposure exhibited strong negative correlations with microalgal biomass (r = −0.62), chlorophyll a (r = −0.63), chlorophyll b (r = −0.52), and the maximum photochemical efficiency of PS II (Fv/Fm, r = −0.73). Mantel’s test (Fig. 5B) further confirmed a robust link between PHMG concentration and Fv/Fm, with photosynthetic efficiency being associated with oxidative stress markers (CAT, MDA) and metabolic products. Structural equation modeling (Fig. 5C) indicated that PHMG was significantly adversely associated with the photosynthetic system of algal cells (path coefficient (λ) = −0.524). The photosynthetic system exerted both positive (λ = 0.711) and negative (λ = −0.471) effects on metabolic and antioxidant systems, while biomass accumulation was positively influenced by metabolic (λ = 0.103), photosynthetic (λ = 0.978), and antioxidant (λ = 0.169) pathways.

## Discussion

### Impact of PHMG exposure on microalgae growth

In this study, we analyzed the effect of different PHMG concentrations on the biomass of microalgae by monitoring the biomass of the measured microalgae, low concentrations of PHMG (below 0.03 mmol/L) may have minimal effects on the microalgae development, whereas higher concentrations (above 0.3 mmol/L) severely hinder their growth. It is noteworthy that long-term exposure of the high PHMG group (R5) produced significant growth inhibition of microalgae. This may be related to the impaired photosynthesis system of microalgae. The maximum Fv/Fm of PS II was consistently low throughout the experimental period, suggesting that the energy conversion efficiency of photosynthesis was severely limited. The results of this study are similar to those of Wanwan, Yan & Weihao (2011). Microalgae gradually adapted to the stress environment after a certain time of disinfectant stress with trichlorohydroxydiphenyl ether (0.18‰∼0.3‰) as the main inhibitory ingredient, *i.e*., showed a certain degree of environmental adaptability, and showing a clear concentration-effect relationship (Wanwan, Yan & Weihao, 2011). It has been shown that antibacterial agents commonly used in medicine such as levofloxacin (LVFX) and clarithromycin (CAM) are both highly toxic to microalgae. Microalgae growth could not be observed at LVFX concentrations above 2,500 mg/L or CAM concentrations above 25 mg/L (Yamashita et al., 2006). By comparing the inhibitory concentrations of these two antibacterial agents with the PHMG concentrations in the high exposure groups (R3: 47.17 mg/L, R4: 94.33 mg/L, and R5: 471.67 mg/L) of this experiment, it can be concluded that PHMG, as an antibacterial agent, exhibits a certain level of toxicity to algae in the aquatic environment.

### Impact of PHMG on the photosynthetic system of microalgae

This study analyzed the effects of different concentrations of PHMG on the photosynthetic system of microalgae by monitoring changes in chloroplast pigments and chlorophyll fluorescence parameters. Algal cells depend on chloroplasts for energy production through photosynthesis (Zhao et al., 2019; Wang et al., 2022a). Exposure to 0.3 and 0.6 mmol/L of PHMG inhibited microalgae photosynthesis, leading to lower chlorophyll *a* and chlorophyll *b* contents throughout the growth period, although the continuous chlorophyll synthesis trend in microalgal cells remained unchanged. This study demonstrated the resilience and adaptability of microalgae when exposed to the moderate concentration (0.03 mmol/L) of PHMG.

The maximum photochemical efficiency of PS II, indicated by Fv/Fm, is a crucial parameter for assessing the photosynthesis capacity to convert light energy into chemical energy during the photochemical phase, in contrast to changes in the chloroplast content (Yang et al., 2010). Specifically, exposure to 3.0 mmol/L of PHMG resulted in a continuously low Fv/Fm ratio of 0.02–0.08 throughout the entire observation period. The monitoring results showed that, similar to the chloroplast content, the maximal photochemical efficiency of PS II (Fv/Fm) similarly responded to PHMG exposure (He et al., 2022). This is consistent with the results of He et al. (2022). The changes in Fv/Fm values were not significant at relatively low concentrations of chlorine-derived metformin by-products, however, the inhibition of photosynthesis system II was irreversible at high concentrations.

### Impact of PHMG on metabolites of microalgae

EPS are biopolymers secreted by microbes during the growth and metabolism, mainly consisting of large organic molecules including polysaccharides and proteins (Russel et al., 2020; Wang et al., 2022c; Ou et al., 2023). EPS, a fundamental metabolic byproduct of microalgae, reacts to antibacterial drugs such as PHMG by exhibiting changes in its composition.

Microalgal cells, which are larger than bacterial cells and possess cell walls primarily composed of cellulose, exhibit the increased resistance to antibacterial drugs like PHMG (Ryan, Ralph & McMinn, 2004). Therefore, no notable effects were observed during the first 1–2 days of exposure. However, the prolonged exposure resulted in the accumulation of PHMG in cells, leading to damage to microalgal cells and a subsequent reduction in the release of metabolic products. Additionally, higher exposure concentrations could further exacerbate the differences between EPS and the control group. When the microalgae were exposed to a high concentration of 3.0 mmol/L PHMG, the secretion of EPS consistently declined over time. Excessively high concentrations of PHMG had a severe toxic impact on microalgal cells, significantly hindering the growth metabolism of the microalgal system.

### PHMG’s influence on the antioxidant system

CAT, SOD, and MDA are critical indicators for microalgal cells’ response to oxidative stress. When exposed to the PHMG-induced stress, microalgae generated excessive reactive oxygen species (ROS), resulting in oxidative damage and MDA release. As a result, microalgae enhanced the activity of antioxidant enzymes such as CAT and SOD to reduce ROS levels, thereby protecting cellular components like proteins, lipids, and DNA from oxidative damage. The study shows that the SOD levels in R. subcapitata exposed to 6.2 to 30 mg/L of exposure of erythromycin trended to increase (Aderemi et al., 2018; Ma et al., 2021). It is worth noting that this exposure concentration is much lower than the PHMG exposure concentration in this experiment, and it can be speculated that the ecological risk of PHMG to algae in the aquatic environment might be relatively low. The temporal dynamics of microalgae responses to PHMG-induced oxidative stress caused by PHMG highlighted the effectiveness of these biomarkers in promptly detecting the cellular harm and assessing environmental risks associated with PHMG exposure.

PHMG (polyhexamethylene guanidine) is a cationic polymer that exerts its biocidal effects primarily by disrupting microbial cell membranes. In this study, we observed that PHMG exposure caused significant damage to the photosynthetic apparatus of microalgae, particularly impairing Photosystem II efficiency and reducing chlorophyll content. Additionally, PHMG induced oxidative stress in microalgae, as evidenced by increased levels of antioxidant enzymes (*e.g*., CAT, SOD) and malondialdehyde (MDA). These findings suggest that PHMG’s mode of action involves both direct membrane disruption and secondary oxidative stress.

### Microalgae’s reaction pathways and processes when exposed to PHMG

PHMG exposure disrupts microalgal physiological functions through dual mechanisms: on one hand, it stimulates the CAT and MDA in microalgae, while on the other hand, it severely inhibits biomass accumulation and photosynthetic system functionality. SEM further indicated that photosynthetic system impairment (λ = −0.524) regulated metabolism (λ = 0.711) and the antioxidant system (λ = −0.471) through energy allocation, whereas biomass accumulation predominantly depended on photosynthetic efficiency (λ = 0.978). Thus, PHMG primarily inhibited microalgal biomass by regulating the microalgal antioxidant system, reducing extracellular polymer secretion, and altering the efficiency of light energy conversion in microalgae.

## Conclusion

This work conducted the experimental analyses on impacts of various concentrations of polyhexamethylene guanidine (PHMG) exposure on the physiological and biochemical traits of microalgae, along with their underlying mechanisms. The key conclusions are as follows:

(1) PHMG had a notable impact on microalgae that increased with the dosage. Under concentrations equal to or exceeding 3 mmol/L, PHMG significantly hindered the growth of microalgae, resulting in reduced biomass, whilst concentrations as low as 0.03 mmol/L exhibited minimal effects on microalgae growth.

(2) PHMG inhibited the production of chlorophyll *a*/*b* in microalgae and decreased the maximal efficiency of light energy conversion in Photosystem II (PS II). The inhibitory effect on the photosynthetic system intensified with increasing PHMG exposure to microalgae adversely affected the metabolic processes and decreased the levels of polysaccharides and proteins in extracellular polymeric substance (EPS) due to the cumulative damage. When exposed to PHMG, microalgal cells promptly activated the antioxidant defense mechanisms to eliminate reactive oxygen species (ROS) by producing more catalase (CAT) and superoxide dismutase (SOD) to combat oxidative stress, which consequently elevated the malondialdehyde (MDA) content.

(3) The structural equation modeling (SEM) analysis showed that PHMG exposure primarily hindered the growth of microalgae by impacting their photosynthetic system, leading to disturbances in metabolism and antioxidant systems.

(4) This study provides valuable insights into the effects of PHMG on *C. vulgaris*, enhancing our understanding of its potential impacts on aquatic ecosystems. The results indicate that PHMG concentrations could pose ecological risks, particularly in environments where disinfectants are widely used, contributing to the growing body of knowledge regarding the environmental impact of such substances.

## Supplemental Information

10.7717/peerj.19553/supp-1 Supplemental Information 1 Raw data.

## Additional Information and Declarations

### Data Availability

The following information was supplied regarding data availability:

The raw data is available in the Supplemental File.
